# Mitochondria-ER contact sites expand during mitosis

**DOI:** 10.1016/j.isci.2024.109379

**Published:** 2024-03-01

**Authors:** Fang Yu, Raphael Courjaret, Lama Assaf, Asha Elmi, Ayat Hammad, Melanie Fisher, Mark Terasaki, Khaled Machaca

**Affiliations:** 1 Calcium Signaling Group, Research Department, Weill Cornell Medicine Qatar, Education City, Qatar Foundation, Doha, Qatar; 2 Department of Physiology and Biophysics, Weill Cornell Medicine, New York, NY, USA; 3 College of Health and Life Science, Hamad bin Khalifa University, Doha, Qatar; 4 Department of Cell Biology, UConn Health, 263 Farmington Ave, Farmington, CT 06030, USA

**Keywords:** Biological sciences, Cell biology, Natural sciences

## Abstract

Mitochondria-ER contact sites (MERCS) are involved in energy homeostasis, redox and Ca^2+^ signaling, and inflammation. MERCS are heavily studied; however, little is known about their regulation during mitosis. Here, we show that MERCS expand during mitosis in three cell types using various approaches, including transmission electron microscopy, serial EM coupled to 3D reconstruction, and a split GFP MERCS marker. We further show enhanced Ca^2+^ transfer between the ER and mitochondria using either direct Ca^2+^ measurements or by quantifying the activity of Ca^2+^-dependent mitochondrial dehydrogenases. Collectively, our results support a lengthening of MERCS in mitosis that is associated with improved Ca^2+^ coupling between the two organelles. This augmented Ca^2+^ coupling could be important to support the increased energy needs of the cell during mitosis.

## Highlights

• Mitochondria-ER contact sites (MERCS) increase in length in mitosis
• MERCS lengthening confirmed using ultrastructure, 3D rendering, and junction markers
• Ca^2+^ transfer from the ER to the mitochondria is enhanced in mitosis

## Graphical abstract

**Figure:**
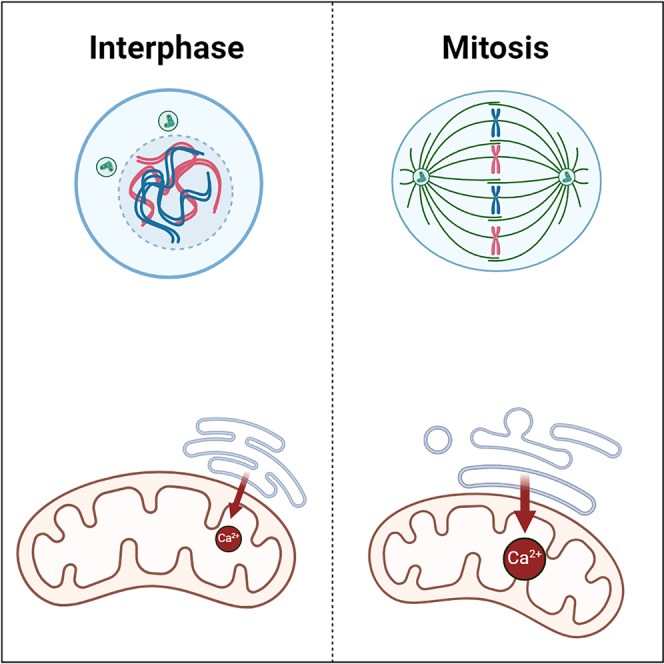

## Introduction

Membrane contact sites (MCS) are close appositions between membrane-bound organelles that form specialized signaling hubs for inter-organelle communications, including, but not limited, to non-vesicular lipid transfer and Ca^2+^ signaling.^1^^,^^2^^,^^3^ The exchange of ions and lipids at MCS argues for a role in regulating metabolic fluxes. The functional importance of MCS is highlighted by their emerging roles in multiple diseases including neurodegenerative disorders^4^ and cancer.^5^

The endoplasmic reticulum (ER) is the largest intracellular organelle and consists of an extensive network of tubules and cisternae distributed throughout the cell. The ER makes contacts with most other organelles including the mitochondria.^6^ MCS between the mitochondria and smooth ER (mitochondria-ER contact sites [MERCS]) are usually 10–50 nm thick and are stabilized by various tethers that form complexes to bridge the junctions between the two organelles.^3^^,^^7^ MERCS tethers include inositol 1,4,5-trisphosphate receptors, voltage-dependent anion channels, the heat shock protein GRP75, mitofusin 2, VAPB, and PTPIP51.^3^^,^^8^ MERCS have been studied extensively and shown to be involved in Ca^2+^ signaling, energy homeostasis, redox biology, proteostasis, inflammation, and autophagy.^3^^,^^9^^,^^10^^,^^11^

Membrane-bound intracellular organelles regulate most cellular functions, including growth, survival, movement, and interactions with the cell’s environment. Cell division generates two daughter cells where each must be able to support these vital functions. Therefore, cell division requires mechanisms for proper inheritance of cellular organelles among the two daughter cells,^12^ particularly because most cellular organelles cannot be generated *de novo* and need to grow from existing seed organelles.

Practically all cellular organelles remodel during mitosis, including the ER and mitochondria. The ER, which is continuous with the nuclear envelope, reorganizes during mitosis with the associated breakdown of the nuclear envelope to allow for chromosome separation.^13^^,^^14^^,^^15^^,^^16^ The ER in interphase consists of a network of cisternae or sheets closer to the nucleus and interconnected tubules toward the cell periphery. In mitosis, the ER restructures into ring-like structures around the cell cortex; however, there is debate in the literature regarding the specific ER morphology in mitosis, whether it consists primarily of sheets with low curvature or more branched tubules or the so called fenestrated sheets with a higher curvature potential.^17^^,^^18^^,^^19^^,^^20^ The reasons for these discrepancies are not entirely clear but may be due to a combination of cell type differences, technical issues such as fixation artifacts for ultrastructural analyses and difficulties in obtaining satisfactory resolution in the z axis, and the transient nature of mitosis.^17^^,^^18^^,^^19^^,^^20^

The mitochondria also remodel during mitosis through complex processes that involve structural changes and alterations to their association with the cytoskeleton. In interphase, mitochondria form an interconnected network of dynamic tubules, the structure of which is maintained by a balance between mitochondrial fission and fusion. Mitochondrial fission depends on the activity of the dynamin-related GTPase Drp1, which localizes to the outer mitochondrial membrane. During mitosis, mitochondria fragment in a process that requires the phosphorylation of Drp1 by the master mitotic kinase Cdk1-cyclin B.^21^ Drp1 regulation and phosphorylation during mitosis depends on the small Ras-like GTPase RALA and its effector RALBP1 and the activity of the cell cycle kinase Aurora A.^22^ To facilitate their partitioning during mitosis, mitochondria dissociate from the microtubule network following the shedding of the microtubule motor proteins, kinesin and dynein.^23^ Instead, mitochondria associate with the growing tips of microtubules in mitosis through interaction between the mitochondrial protein Miro and the cytoskeletal-associated protein Cenp-F.^24^ This interaction supports proper mitochondrial distribution in mitosis. Furthermore, mitochondria partitioning during mitosis was shown to require myosin-XIX, thus arguing for an important role for the actin cytoskeleton.^25^ This is supported by the recent findings of an active role for actin cables in mitochondrial inheritance in mitosis.^26^ Actin forms a subcortical mesh of cables that scaffolds the ER and mitochondria, and further engulfs mitochondria to mediate their comet-like movement and randomization in the mother cell thus supporting proper inheritance to the daughter cells.^26^

In addition, mitochondria are the energy source for the cell, so they must maintain their functionality as the energy demands during cell division are elevated.^27^^,^^28^^,^^29^ Mitochondrial respiration is increased during mitosis by enhancing oxidative phosphorylation through CDK1 phosphorylation of different subunits in complex I.^29^ In addition, a Ca^2+^ transient in metaphase has been shown to be important for maintaining cellular energy levels in mitosis through a mitochondrial calcium uniporter (MCU)-dependent process.^27^ This shows that mitochondrial Ca^2+^ uptake is important for mitosis progression. With the ER being the primary source of intracellular Ca^2+^ release and with MERCS being the preferred site for ER to mitochondria Ca^2+^ transfer, these findings suggest a role for MERCS in mitosis.

However, the fate of MERCS in mitosis has not been explored. Here, we show using multiple approaches that MERCS expand during mitosis and that this expansion is associated with more efficient ER-mitochondrial Ca^2+^ coupling resulting in enhanced activation of matrix dehydrogenases.

## Results and discussion

### Thin-section EM

**Figure 1:**
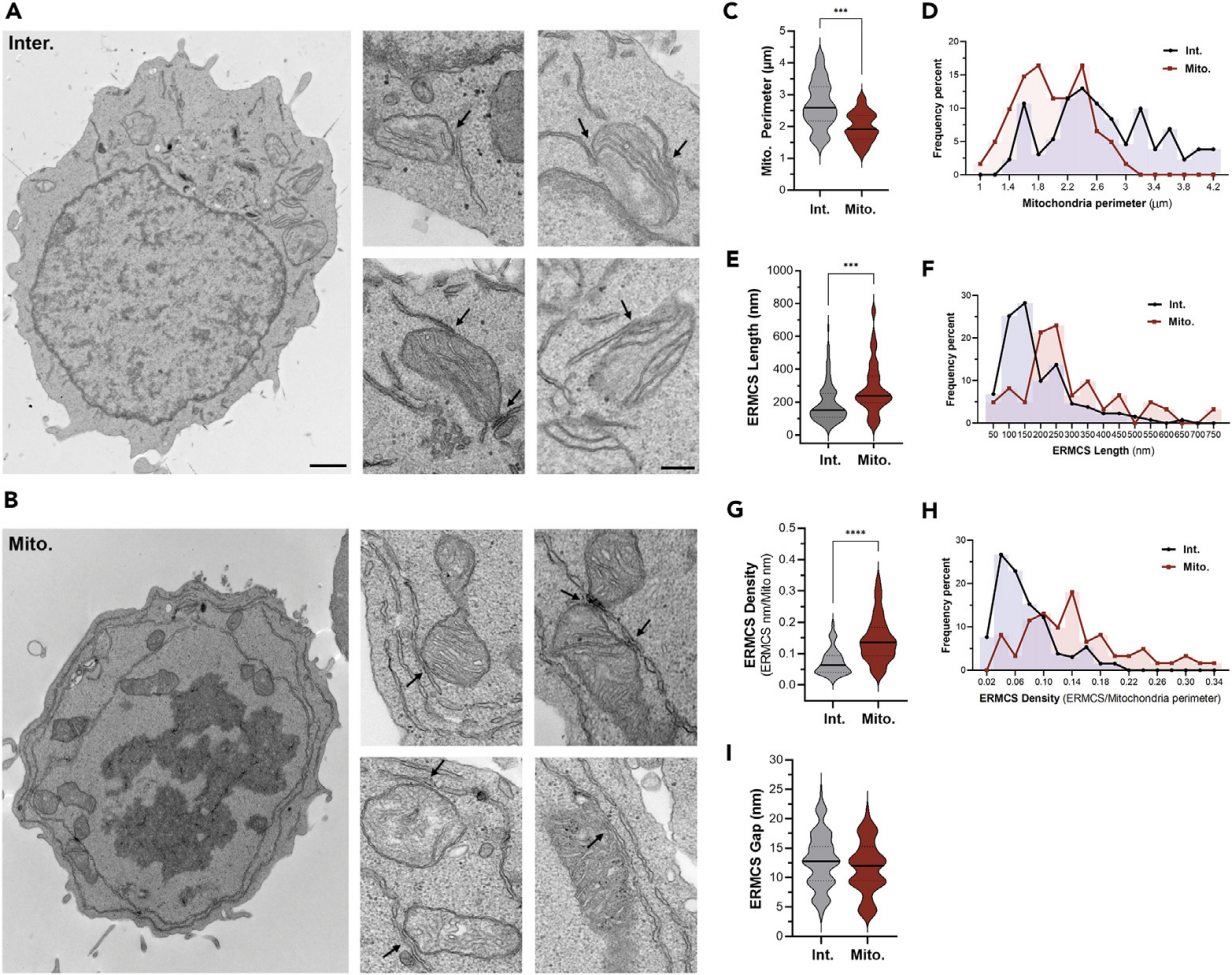
TEM analysis of MERCS in interphase (Inter.) and mitotic (Mito.) Jurkat cells (A and B) Representative TEM images from Inter. (A) and Mito. (B) cells. Black arrows indicate the MERCS defined as close contacts between the ER and mitochondrion at ≤30 nm. A representative whole cell is shown at low magnification (scale bar 1 μm), with several zoomed-in regions (scale is 250 nm) containing MERCS (arrows in A and B). (C, E, G, and I) Quantification of mitochondrial perimeter (C), MERCS length (E), MERCS density (G), and MERCS gap (I) in interphase and mitotic cells (Mean ± SEM, unpaired two tailed t test, n = 61–131, ∗∗∗p ≤ 0.001, ∗∗∗∗p ≤ 0.0001). (D, F, and H) Frequency histogram of mitochondrial perimeter (D), MERCS length (F), and MERCS density (H).

We first assessed MERCS during interphase and mitosis at the ultrastructural level using thin-section electron microscopy (EM) in Jurkat T lymphocytes. We chose Jurkat cells due to their relatively less complex intracellular organelle organization compared to other cells (Figures 1A and 1B). As is apparent in the example images in Figures 1A and 1B, mitosis is associated with dramatic remodeling of organelles: nuclear envelope breakdown, chromosome condensation, and ER remodeling where the ER transitions from a broadly distributed network throughout the cell in interphase to cortically enriched rings in mitosis (Figure 1B). We have previously shown that ER-plasma membrane contact sites (ERPMCS) are lost in mitosis.^30^ So, we were interested in assessing whether this downregulation also applies to MERCS. We reasoned that if organelles are to separate between the two daughter cells the less MCS the better, as it would decrease co-segregation and potential membrane damage given the forces acting in mitosis to separate organelles. However, a scan of MERCS in mitosis argues for the opposite (Figures 1A and 1B). As shown in the multiple examples in Figure 1B, MERCS in mitosis appear to extend along longer lengths of the mitochondria as compared to interphase cells (Figure 1A). In some cases, the ER is in close apposition through the entire length of a mitochondrion (Figure 1B, lower right panel, arrow).

To confirm these observations, we quantified MERCS length, density, and gap as well as mitochondrial perimeter from EM thin sections. These quantifications were performed independently by two individuals, which were blinded to each other’s findings and produced similar results. Mitochondrial perimeter measurements reveal a smaller average perimeter in mitosis (1.98 ± 0.059 μm) as compared to interphase (2.67 ± 0.065 μm) (Figure 1C). This was associated to a shift in the histogram distribution of the population toward perimeters smaller than 3 μm (Figure 1D). These observations are consistent with the previously documented mitochondrial fragmentation in mitosis.^21^^,^^22^ However, because these measurements were performed on thin sections they cannot be taken as independent validation for mitochondrial fission as a smaller perimeter in a thin section could be due to a smaller diameter in a cross section across the mitochondria.

**Figure 2:**
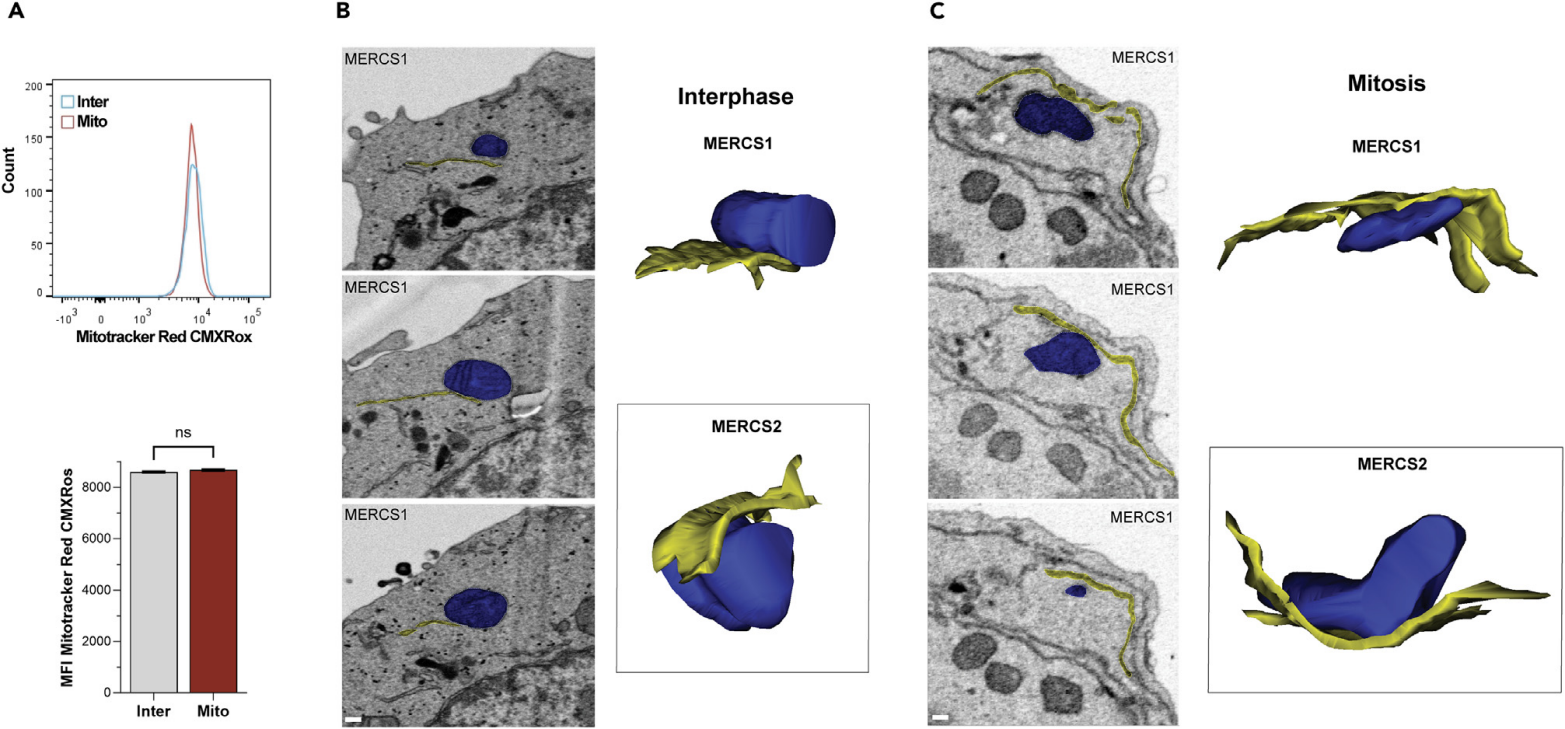
3D reconstruction of MERCS (A) Mitochondrial volume estimates in interphase (Inter) and naturally occurring mitotic (Mito) Jurkat cells were measured using MitoTracker Red. Representative flow cytometry plots (top) and summary data (down) are shown (mean ± SEM, n = 4). (B and C) Representative 3D reconstruction in interphase (B) and mitosis (C) from serial EM of ER (yellow) and mitochondria (blue). Example slices from the serial sections are shown on the left with the associated tracing of the ER and mitochondria. Scale bar is 50 nm. Images from the beginning, middle, and end of the stack used for the 3D reconstruction are shown. The top 3D rendering is from the stack example shown on the right (MERCS1), whereas the bottom one is from another ER-mitochondrial contact (MERCS2).

Importantly, quantification of MERCS length shows a significant increase in mitosis (284.4 ± 20 nm) as compared to interphase (189.3 ± 9.9 nm) (Figure 1E). This increase is coupled to a shift in the histogram toward MERCS above 200 nm in length as compared to interphase MERCS whose distribution shows a peak in the 100–150 nm range (Figure 1F). This shows that the majority of MERCS are longer in mitosis compared to interphase (Figure 1F). We further quantified MERCS density as the percent of the mitochondrial perimeter occupied by MERCS as this could be functionally significant in terms of coupling between the two organelles. As expected from a smaller perimeter and longer MERCS in mitosis, MERCS density is significantly higher in mitosis (0.145 ± 0.0093) as compared to interphase (0.073 ± 0.0036) (Figures 1G and 1H). We did not detect a significant change in MERCS gap distance between the two stages of the cell cycle (Figure 1I). These results show that MERCS length and density increase during mitosis. These increases are not associated with dramatic changes in total mitochondrial volume during mitosis, as revealed by crude assessment of mitochondrial volume using Mitotracker (Figure 2A), consistent with previous estimates.^31^

### Serial EM and 3D reconstruction

The thin-section EM analyses argue for an increase in MERCS length during mitosis. We were however concerned that these measurements may be somewhat biased by the selection and angle of the sections as they do not provide a full 3D view of the entire MERCS, despite the relatively large number of MERCS assessed (131 in interphase and 61 in mitosis). To confirm and expand the thin-section analyses of MERCS remodeling in mitosis, we performed serial EM sectioning on three interphase and three mitotic cells across the entire cell and used the resultant stacks to trace MERCS. Tracing of MERCS in interphase and mitosis was performed using Reconstruct software as shown in Figures 2B and 2C by three independent individuals with similar results. We show examples of two 3D renditions each from interphase (Figure 2B) and mitosis (Figure 2C). The 3D reconstructions support the quantifications from thin sections as they show increased interactions between the ER and mitochondria in mitosis (Figures 2B and 2C). In interphase, the edge of the ER comes in close contact with the mitochondria to form MERCS (Figure 2B). In contrast, the mitochondria in mitosis appear to embed within an ER basket, resulting in the longer more expansive MERCS (Figure 2C). As these experiments allow for assessment of the entire MERCS volume at the ultrastructural level, they confirm the thin-section EM quantifications and support the conclusion that MERCS expand during mitosis.

### MERCS expand in mitosis in different cell types

T cells are a useful cell model to analyze MERCS at the ultrastructural level due to their small size, large volume occupied by the nucleus, and relatively low organellar complexity. However, T cells are specialized, so we were interested in determining whether the increased MERCS length in mitosis occurs in other cell types. Furthermore, our assessment of MERCS so far has been limited to cells fixed and processed for EM, which may introduce artifacts in MERCS structure. To address these concerns, we resorted to a higher throughput approach to assess MERCS using the split GFP reporter (sGFP) with two proteins each containing a complementary GFP fragment expressed separately in the ER and mitochondrial membrane. With these constructs, full-length functional GFP is formed only when the ER and mitochondrial membranes are in close proximity as within MCS, resulting in a fluorescent signal only at MERCS.^32^ Because expression of these markers at high levels may be toxic and because transient transfections resulted in a large spectrum of expression, which would affect MERCS quantification, we generated stable cell lines expressing the sGFP-MERCS reporter at low levels in both HeLa and HEK293 cells.

**Figure 3:**
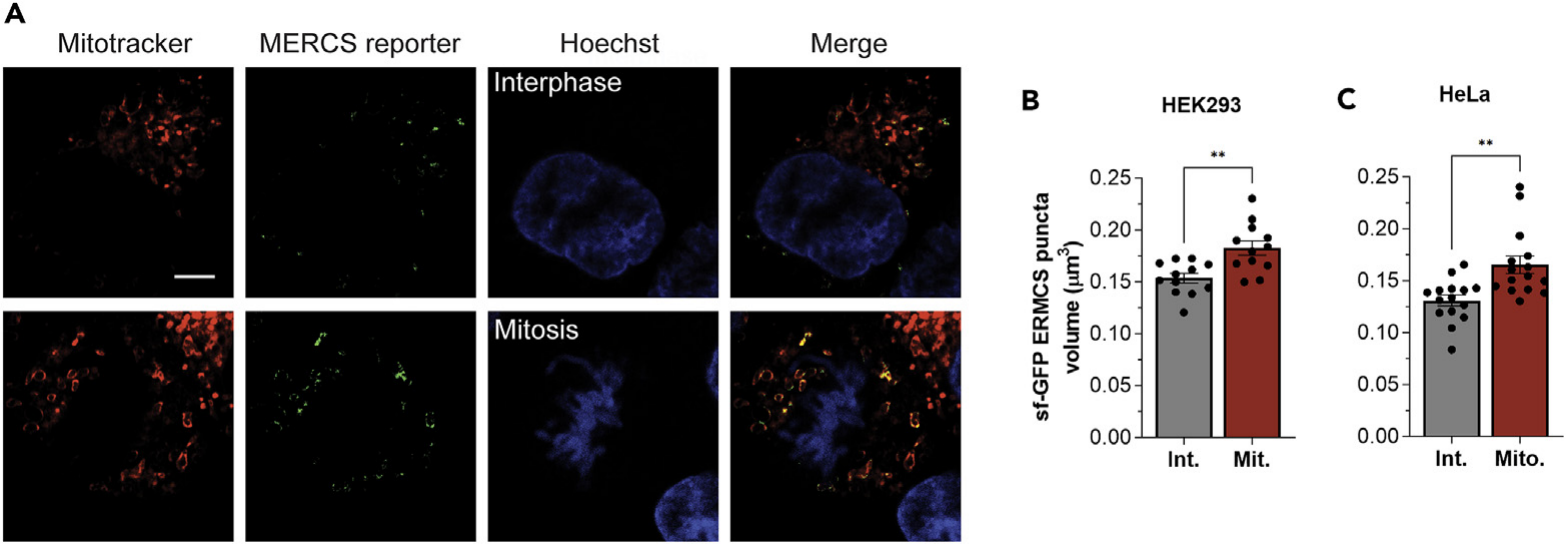
MERCS increase in mitotic HEK293 and HeLa cells (A) Representative confocal images from stable HEK293 cells expressing the MERCS reporter either in interphase or naturally occurring mitosis. Cells were stained with MitoTracker deep red to identify mitochondria and Hoechst to differentiate cells in interphase from those in mitosis. Scale bar 5 μm. (B and C) Quantification of MERCS volume after 3D rendering using Imaris software in HEK293 (B) and HeLa (C) cells stably expressing the split GFP MERCS reporter. (B) Mean ± SEM, unpaired two tailed t test, n = 12, p = 0.0019. (C) Mean ± SEM, unpaired two tailed t test, n = 15, ∗∗p ≤ 0.01.

Cells were stained with Hoechst to identify mitotic cells and Mitotracker to confirm localization of the sGFP signal to mitochondria (Figure 3A). Because we wanted to assess total MERCS across the entire cell to minimize error in assessing the fluorescence signal from individual confocal sections, we collected a confocal z stack across the cell. We rendered the GFP signal from MERCS across the z stack using Imaris software and quantified the volume of the resultant spots in both HEK293 and HeLa cells (Figures 3B and 3C). In both cell types, we observed a significant increase in the volume of GFP-marked MERCS in mitosis as compared to interphase (Figures 3B and 3C). These results are consistent with the increased MERCS length observed in the ultrastructural studies. A limitation in the sGFP experiments is the different shape of the cells in interphase which are adherent, compared to those in mitosis which are rounded. Cell rounding in mitosis may affect the cell’s refractive index and cause spherical aberrations. As discussed previously, the EM experiments as well suffer from technical limitations primarily from a potential effect of fixation on MERCS structure. However, three different experimental approaches in different cell lines support the conclusion that MERCS expand in mitosis: thin-section EM, serial EM coupled to 3D reconstruction, and live-cell imaging of tagged MERCS. This augments our confidence in the conclusion that MERCS length and extent increase in mitosis.

### MERCS expansion in mitosis is associated with enhanced Ca^2+^ transfer to mitochondria

As discussed previously, mitochondrial respiration has been shown to increase in mitosis^27^^,^^29^ presumably to meet the increased energy demands of the dividing cell. Mitochondrial Ca^2+^ transients have been shown to be important to support the cell’s energy demands in mitosis. Given the close apposition of the two organelle membranes at MERCS, they are preferred sites for efficient Ca^2+^ transfer to activate Ca^2+^-sensitive matrix dehydrogenases.^33^^,^^34^ Furthermore, the MCU channel in the inner mitochondrial membrane has been shown to be important for the mitochondrial Ca^2+^ transients in mitosis.^27^ We therefore wondered whether the lengthening of MERCS in mitosis increases coupling between the two organelles.

**Figure 4:**
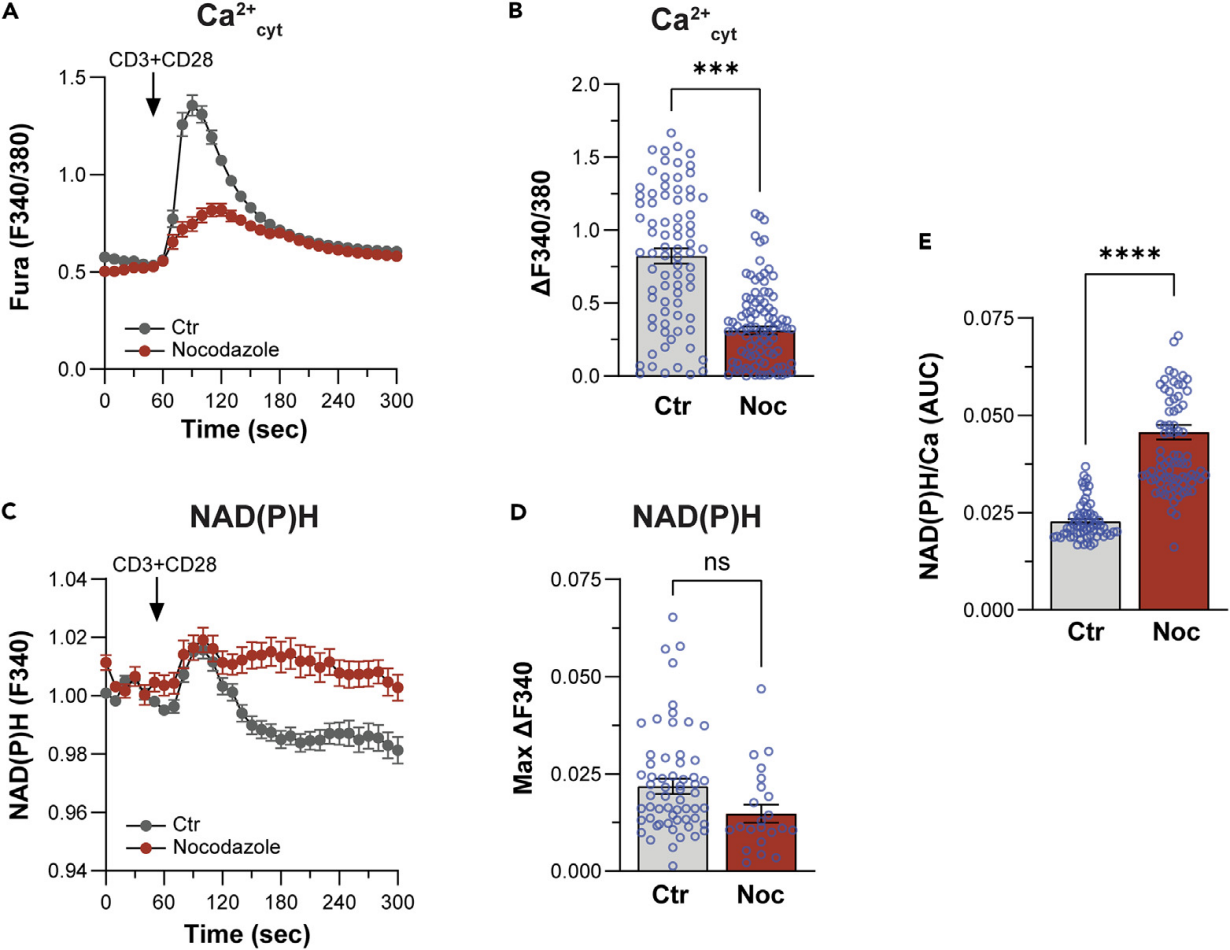
Intracellular Ca^2+^ signals and NAD(P)H levels in interphase and mitosis (A) Cytoplasmic Ca^2+^ (Ca^2+^_cyt_) time course in response to CD3/CD28 stimulation in Jurkat T cells in asynchronous or mitotic (nocodazole) cells. (B) Quantification of the peak Ca^2+^_cyt_ response. Mean ± SEM, unpaired two tailed t test, n = 79–104, ∗∗∗p ≤ 0.001. (C) Time course of the NAD(P)H autofluorescence (measured at 340 nm) induced by CD3/CD28 stimulation is asynchronous and mitotic cells. (D) Quantification of the peak NAD(P)H autofluorescence. Mean ± SEM, unpaired two tailed t test, n = 23–61. (E) Coupling between the ER and the mitochondria quantified as the ratio between the average area under the curve (AUC) of the (NAD(P)H signal and the individual AUC of the Ca^2+^_cyt_ signal induced by CD3/CD38 stimulation. Mean ± SEM, unpaired two tailed t test, n = 66–86, ∗∗∗∗p ≤ 0.0001.

To assess Ca^2+^ coupling between the ER and mitochondria during cell division, we wanted to directly record mitochondrial Ca^2+^ transients in Jurkat T cells. So we expressed ER and mitochondria-targeted CEPIA Ca^2+^ sensors to concurrently record ER and mitochondria Ca^2+^ levels as previously described.^35^ Unfortunately, we were not able to detect mitotic cells that express both sensors, which were only efficiently expressed in interphase cells. We therefore resorted to a less invasive approach to assess ER-mitochondria coupling in Jurkats, using endogenous NAD(P)H autofluorescence as a readout for Ca^2+^-dependent activation of mitochondrial dehydrogenases^36^ (Figure 4). NADH is autofluorescent and acts as an electron donor for the electron transport chain in the mitochondria. Increased mitochondrial Ca^2+^ activates these dehydrogenases resulting in higher NADH levels, which can be used as a surrogate marker of mitochondrial Ca^2+^-dependent activation of respiration.^34^ Cross-linking the T cell receptor (TCR) using anti-CD3 and anti-CD28 antibodies in Jurkat T cells stimulates PLC signaling and Ca^2+^ release from stores (Figure 4A). The Ca^2+^ release transients were of significantly higher amplitude in interphase compared to mitosis (Figures 4A and 4B). We recorded in parallel NAD(P)H autofluorescence in interphase and mitosis (Figure 4C). NAD(P)H autofluorescence was similar following TCR stimulation in both stages of the cell cycle (Figures 4C and 4D).

We used the ratio of the NAD(P)H autofluorescence to that of the Ca^2+^ signal to assess ER-mitochondria Ca^2+^ coupling (Figure 4E). This ratio provides a measure of the efficacy of the Ca^2+^ release transient in activating mitochondrial dehydrogenases. ER-mitochondria Ca^2+^ coupling was significantly enhanced in mitosis, where matrix dehydrogenases were stimulated more effectively in response to a smaller Ca^2+^ transient (Figure 4E). This argues that the expansion of MERCS in mitosis is functionally important to enhance Ca^2+^ coupling and support respiration.

**Figure 5:**
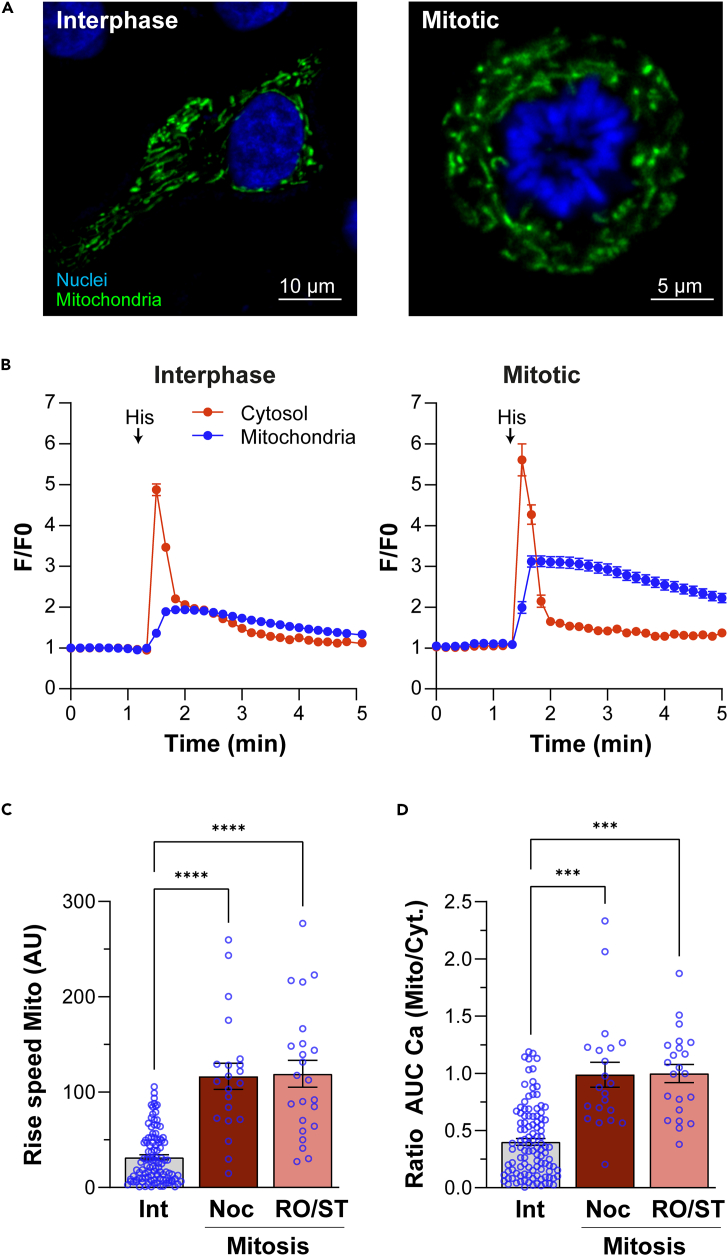
ER-mitochondria Ca^2+^ coupling in HeLa cells in interphase and mitosis (A) Airy Scan images of the nuclei (Hoechst, blue) and mitochondria (CEPIA2*mt*, green) in interphase and mitosis HeLa cells. (B) Time course of the Ca^2+^ response in the cytosol and mitochondria of HeLa cells in interphase and mitosis following stimulation with 100 μM histamine (His) (mean ± SEM, n = 44–115, ∗∗∗∗p ≤ 0.0001). (C) Rise speed of the Ca^2+^ signal in the mitochondria from baseline to peak in interphase and mitotic cells (mean ± SEM, ANOVA Dunnett correction, n = 21–107). (D) Coupling between the ER and the mitochondria quantified as the ratio between the integer of the Ca^2+^ signal in the mitochondria and in the cytosol induced by histamine during the first minute (area under the curve) (mean ± SEM, ANOVA Dunnett correction, n = 21–109, ∗∗∗p ≤ 0.001). Noc: nocodazole; RO/ST: RO-3306 and STLC

NAD(P)H fluorescence does not explicitly report mitochondrial Ca^2+^ but rather provides a functional surrogate for it. We wanted to expand the NAD(P)H studies by simultaneously recording mitochondrial and cytosolic Ca^2+^ in response to agonist-driven Ca^2+^ release from stores. Such experiments were technically not possible in Jurkat cells as they did not tolerate electroporation to express a mitochondrial Ca^2+^ sensor and drug treatment to enrich them in mitosis. We therefore used HeLa cells transfected with the mitochondrial Ca^2+^ sensor CEPIA2*mt* and loaded with Calbryte 590 to record mitochondrial and cytosolic Ca^2+^ transients, respectively. We enriched mitotic cells using two different approaches to rule out any drug nonspecific effects; we employed either nocodazole or a combination of a Cdk1 inhibitor (RO-3306) and the kinesin motor inhibitor (STLC).^37^ We were able to detect mitotic cells that express CEPIA2*mt* (Figure 5A).

The application of histamine induced a fast and transient ER Ca^2+^ release spike in both interphase and mitotic cells (Figure 5B, cytosol). This was followed by a slower rise in mitochondrial Ca^2+^, which was significantly larger in mitotic as compared to interphase cells (1.02 ± 0.07, n = 115; vs. 2.06 ± 0.12, n = 44; p < 0.001, unpaired t test) (Figure 5B). Since the amplitude of the cytosolic Ca^2+^ signal was slightly but significantly larger in mitotic as compared to interphase cells (5.26 ± 0.30, n = 44; vs. 4.12 ± 0.11, n = 115; p < 0.001, unpaired t test), we quantified the coupling between ER Ca^2+^ release and the rise in mitochondrial Ca^2+^ using two approaches. We quantified the speed of the Ca^2+^ rise in the mitochondria immediately following the cytosolic transient (Figure 5C), and we normalized the mitochondrial signal to the cytosolic Ca^2+^ transient on a per cell basis (Figure 5D). We observe a significantly faster Ca^2+^ rise in mitotic cells as compared to interphase using either mitotic arrest protocol (Figure 5C). This supports tighter coupling between the two organelles in terms of Ca^2+^ transfer to the mitochondria in mitosis. Similarly, quantification of the ratio of the integer (area under the curve) of the mitochondrial Ca^2+^ transient to that of the cytosolic Ca^2+^ rise in the first minute after histamine stimulation reveals a significant increase in coupling in mitosis as compared to interphase (Figure 5D). We quantified the ratio of the Ca^2+^ signals right after Ca^2+^ release to assess Ca^2+^ transfer to the mitochondria and avoid other mitochondrial Ca^2+^ dynamics, such as Ca^2+^ extrusion, that would modulate the Ca^2+^ signal over a longer time frame. Collectively, these findings show that Ca^2+^ is transferred from the ER to the mitochondria more efficiently in mitosis.

The enhanced Ca^2+^ transfer into the mitochondria in mitosis may affect the spatial dynamics of Ca^2+^ signals, which have been historically implicated in mitosis progression.^38^^,^^39^^,^^40^ For example, localized Ca^2+^ transients have been associated with the metaphase-to-anaphase transition,^41^^,^^42^ and have been detected at the centrosomes in mitosis.^43^ Ca^2+^ signals have also been shown to be dispensable for entry into meiosis but required for the transition of the anaphase.^44^^,^^45^ In addition, a localized mitochondrial Ca^2+^ transient mediated by AMPK activation has been shown to be required for mitosis progression and to boost ATP production.^27^ Hence, the increased ER-mitochondrial coupling leading to enhance mitochondrial Ca^2+^ transfer could modulate cytosolic and mitochondrial Ca^2+^ dynamics in mitosis.

In summary, we show using different quantifications, including thin-section EM, serial EM and 3D reconstruction, and MERCS markers, that MERCS expand in mitosis. Furthermore, quantification of mitochondrial dehydrogenase activity as well as direct measurement of mitochondrial Ca^2+^ argue that coupling between the ER and mitochondria is augmented in mitosis. For this study, we used Jurkat T cells for the ultrastructural and coupling analysis, and HeLa cells for the coupling and MERCS marker analysis; the latter analyses were also supported in HEK293 cells. Although the findings from these three cell types support our conclusions, further studies will be required to determine whether MERCS expansion and its associated increased ER-mitochondrial Ca^2+^ coupling in mitosis are universal processes.

During mitosis, the cell undergoes dramatic changes, including organelle remodeling, chromosome condensation, and nuclear envelope breakdown to name a few. These processes are energy intensive and thus require mitochondria to fuel them. The expansion of MERCS in mitosis would support mitochondrial function. In contrast to MERCS, ERPMCS dissociate in mitosis thus removing these privileged communication sites between the ER and PM.^30^ This remodeling is associated with the loss of Ca^2+^ influx through the store-operated Ca^2+^ entry (SOCE) pathway in mitosis.^30^ Therefore, the well-documented crosstalk between SOCE and the mitochondria^46^^,^^47^^,^^48^ will be lost in mitosis. The expansion of MERCS in mitosis may thus be a compensatory mechanism to maintain Ca^2+^-dependent regulation of mitochondrial metabolism in the absence of SOCE. Furthermore, the tighter physical association between the ER and mitochondria in mitosis could support co-segregation of the two organelles to the daughter cells.

### Limitations of the study

We here demonstrate lengthening of MERCS in mitosis that is associated with increased Ca^2+^ coupling between the two organelles. Although the expansion of the MERCS is likely to be the main contributing factor in the increased coupling, we cannot rule out potential contributions from plasticity of the contact sites in terms of protein and lipid composition. Finally, whether this phenomenon of MERCS expansion in mitosis is universal remains to be determined.

## STAR★Methods

### Key resources table

**Table:** 

REAGENT or RESOURCE	SOURCE	IDENTIFIER
**Antibodies**		

Ultra-LEAF Purified anti-human CD3 Antibody (clone OKT3)	BioLegend	Cat. #317325
Purified anti-human CD28 Antibody (clone 28.2)	BioLegend	Cat. #302902
Alexa Fluor 647 Rat anti-Histone H3 (pS28) (Clone HTA28)	BD Biosciences	Cat. #558217

**Chemicals, peptides, and recombinant proteins**		

MitoTracker Red CMXRox	Invitrogen	Cat. #M7512
MitoTracker Deep Red FM	Invitrogen	Cat. #M22426
Nocodazol	Sigma-Aldrich	Cat. #M1404
Paraformaldehyde (PFA)	Sigma-Aldrich	Cat. #30525-89-4
Dulbecco’s Modified Eagle Medium (DMEM)	Gibco	Cat. #11995073
RPMI 1640 Medium	Gibco	Cat. #11875093
Fetal Bovine Serum (FBS)	Gibco	Cat. #16-000-044
Penicillin/Streptomycin	Gibco	Cat. #15140122
NuclearID Red	Enzo Life Sciences	Cat. #ENZ-52406
Fixable Viability dye eFluor 506	eBioscience	Cat. #65-0866-14
Puromycin	Gibco	Cat. #A1113803
Blasticidin	Gibco	Cat. #A1113903
Fura-2/AM	Invitrogen	Cat. #F1221
Calbryte 590 AM	AAT Bioquest	Cat. #20700
Histamine	Sigma-Aldrich	Cat. #H7125
Ro-3306	Selleckchem	Cat. #S7747
S-Trityl-L-cysteine (STLC)	TOCRIS	Cat. #2191
DAPI	Invitrogen	Cat. #D1306
Hoechst33324	Invitrogen	Cat. #H3570

**Experimental models: Cell lines**		

HEK293 (Female)	ATCC	CRL-1573
HeLa (Female)	ATCC	CCL-2
Jurkat, Clone E6-1 (Male)	ATCC	TIB-152
HEK293 exogenous ER-mito MCS reporter	This Paper	N/A
Hela exogenous ER-mito MCS reporter	This Paper	N/A

**Recombinant DNA**		

pLVX -Mitot-spGFP11×2	Yang et al., 2018^32^	N/A
plx304-spGFP1-10-ERt	Yang et al., 2018^32^	N/A
pCMV CEPIA2mt	Addgene. Suzuki et al. 2014^49^	plasmid #58218

**Software and algorithms**		

FLOWJO software	BD	https://www.flowjo.com/
FIJI/Image J	NIH	https://hpc.nih.gov/apps/Fiji.html
ZEN Black software version 2.1	Zeiss	
GraphPad Prism version 9.0.0	GraphPad Software	https://www.graphpad.com/features
Reconstruct software version 1.1	SynapseWeb, Kristen M. Harris, PI	https://synapseweb.clm.utexas.edu/software-0
PTI Easy Ratio Pro system software (version 1.6.1.0.101)	Horiba	https://www.horiba.com/int/scientific/products/detail/action/show/Product/easyratiopro-1604/
Imaris software 9.5.0	Oxford Instruments-Bitplane	https://imaris.oxinst.com/
Clampfit 10.7.03	Molecular Devices	https://www.moleculardevices.com

**Other**		

FACSAria II cell sorter	BD Biosciences	https://www.bdbiosciences.com/content/dam/bdb/marketing-documents/BD-FACSAria-III-Cell-Sorter-Brochure.pdf
Fortessa X20 cell analyzer	BD Biosciences	https://www.bdbiosciences.com/en-eu/products/instruments/flow-cytometers/research-cell-analyzers/bd-lsrfortessa-x-20
JEOL JEM-1400 Transmission Electron Microscope	JEOL	https://www.jeolusa.com/PRODUCTS/Transmission-Electron-Microscopes-TEM/120-kV/JEM-1400Flash
Zeiss LSM 880 confocal microscope	Zeiss	Zeiss.com/microscopy
Olympus IX71 inverted microscope	Olympus	https://www.olympus-lifescience.com/data/olympusmicro/brochures/pdfs/ix71.pdf

### Resource availability

#### Lead contact

Further information about the protocols and requests for resources and reagents should be directed to and will be fulfilled by the lead contact, Khaled Machaca (khm2002@qatar-med.cornell.edu).

#### Materials availability

Cell lines (HEK293 exogenous ER-mito MCS reporter and Hela exogenous ER-mito MCS reporter) generated in this study are available upon request.

#### Data and code availability

• All data reported in this paper will be shared by the lead contact upon request.
• This paper does not report original code.
• Any additional information required to reanalyze the data reported in this paper is available from the lead contact upon request.

### Experimental model and study participant details

#### Cell culture

HEK293, Hela, and Jurkat cells were obtained from ATCC. HEK293 and Hela cells were cultured in DMEM media (Gibco) supplemented with 10% FBS (Gibco), 100U/ml penicillin, and 100μg/ml streptomycin. Jurkat cells were cultured in RPMI 1640 media (Gibco) supplemented with 10% FBS, 100U/ml penicillin and 100μg/ml streptomycin (Gibco). Cells were incubated at 37°C and under 5% CO_2_. For transmission electron microscopy (TEM), Ca^2+^ imaging, and NAD(P)H imaging Jurkat cells were untreated or treated with Nocodazole (100 ng/ml, Sigma) for 18 h to enrich for cells arrested in mitosis.

The stable HEK293 and Hela cell lines with ER-mito MCS reporter expression were generated by lentiviral infection. Briefly, cells were infected with pLVX -Mitot-spGFP11×2 and plx304-spGFP1-10-ERt using standard procedure described elsewhere.^32^ Infected cells were then selected with puromycin and blasticidin. Cells with low to moderate expression levels of the split GFP markers were enriched by a FACSAria II cell sorter (BD Bioscience). We selected cells with lower expression levels of the markers to avoid any potential toxicity.

### Method details

#### Flow cytometry

Mitochondrial volume was measured by loading Jurkat cells with 100 nM Mitotracker red CMXRox (Invitrogen). Cells were harvested with Fixable Viability Dyes eFluor 506 (eBioscience) prior to fixation. Fixed and permeabilized cells were subjected to immunostaining with Alexa Fluor 647 Rat anti-Histone H3 (pS28) (Clone HTA28) antibody, a well-known marker for mitosis (BD Biosciences). The measurement of mitochondrial volume was conducted using a BD Fortessa X20 cell analyzer. Viable naturally occurring mitotic and interphase cells were analyzed. Flow cytometry data were analyzed by the FlowJo software (BD).

#### Confocal microscopy and Image analysis

Cells were cultured on 35-mm glass-bottom dishes (MatTek) and stained at 37°C for 20 min in culture medium with 100 nM Mitotracker deep red to label mitochondria and either Hoechst33324 (Invitrogen) or NuclearID red (Enzo Life Sciences) to label DNA and distinguish cells in interphase or mitosis. To quantify MERCS in interphase and mitotic cells, multi-track acquisition was performed with excitation lines set at 488 nm (for sf-GFP MERCS puncta) at 2% and 561 or 405 nm (for DNA stains) at 2%. Interphase and naturally occurring mitotic cells (either live or fixed with 4% PFA in PBS for 10 minutes at room temperature) were imaged using a Zeiss LSM 880 confocal microscope using a C Plan Apochromat 63×/1.4 Oil DIC UV-VIS-IR M27 objective with the pinhole at 1AU. Z-stacks were collected at 0.443 μm slice interval, stepping through the entire cell. Frame size was set at 792×792 pixels. Images were analyzed using ZEN Black software. MERCS analysis was performed using the Imaris software 9.5.0 (Bitplane). Briefly, to quantify MERCS, the “Spots” creation tool was used to generate a 3D rendition of the MERCS labelled by the split GFP marker with the wizard set at ‘different spot sizes (region growing)’. ‘Estimated Diameter’ was set as 0.400 μm. The background subtraction (local contrast) method was used to threshold sf-GFP MERCS puncta, with the same threshold value across all images. Once the 3D rendition of the labelled MERCS is generated this allows for calculation of the spots volume. The average spot volume of each cell was calculated and used as average volume of sf-GFP MERCS puncta of each cell. We used the same parameters to generate spot sizes among different cells to minimize distortion as any estimation errors due to the spot 3D generation in Imaris would be similar and would at least allow for relative comparison of MERCS volume between interphase and mitosis.

#### Serial EM and 3D reconstruction

To prepare samples for TEM, Jurkat cell pellets were treated with a modified Karmovsky's fix and followed by a secondary fixation in reduced osmium tetroxide as described previously.^30^ The samples were then *En bloc* stained with uranyl acetate and dehydrated with graded ethanol before being embedded in an Epon analog resin. The 65 nm ultrathin sections were contrasted with lead citrate and imaged using a JEM 1400 electron microscope (JEOL, USA, Inc.) operating at 100 kV. Digital images were captured using a Veleta 2 K × 2 K CCD camera (Olympus-SIS). The length, density, gap of MERCS, and perimeter of mitochondria measured using ImageJ. The density of MERCS was quantified as MERCS length divided by the circumference of the mitochondrion.

For serial EM sectioning the methods were as used previously.^50^ Briefly, 50 nm thick sections were collected on kapton tape using an ATUM (automated tape collecting microtome), then imaged by back scatter in a scanning electron microscope. Images were aligned in FIJI/Image J and further aligned using Reconstruct software (version 1.1). Manual tracing and 3D renditions were performed in Reconstruct using the Boissonnat Surface option for 3D representation.

#### Ca^2+^ and NAD(P)H imaging

Ca^2+^ and NAD(P)H imaging of Jurkat cells was performed using a PTI Easy Ratio Pro system (software version 1.6.1.0.101; Horiba Scientific) composed of a DeltaRAMX monochromator and a CoolSnapHQ^2^ camera attached to an Olympus IX71 inverted microscope fitted with a 20x/0.75 lens. For Ca^2+^ imaging the cells were loaded for 30 min with 2 μM Fura2-AM in a Ca^2+^-containing media (composition below) at room temperature and washed once in Ca^2+^-containing or Ca^2+^-free media prior to the experiments. Cells were excited at 340 nm and 380 nm for 100 ms at a 0.1 Hz frame rate and the ratio of the fluorescence intensity at 340/380 measured. For NAD(P)H measurement the cells were excited at 340 nm for 500 ms at 0.1 Hz and emitted light collected through a 420 nm long pass filter (Olympus UMWU2). Jurkat cells were plated on Poly-D-lysine coated coverslips (MatTek) and left for 10 min to allow adhesion to the coverslip. The cells were activated using 1.0 μg/ml of BioLegend Ultra-LEAF™ Purified anti-human CD3 Antibody (clone OKT3) and anti-human CD28 Antibody (clone 28.2). For Ca^2+^ imaging in HeLa cells the cells were transfected for 5 hours with the mitochondria Ca^2+^ indicator CEPIA2*mt*^35^^,^^51^^,^^49^ using lipofectamine 2000. To induce mitotic arrest cells were treated overnight with Nocodazole (100 ng.ml^-1^) or for 5 hours with a combination of the kinesin motor inhibitor (S-Trityl-L-cysteine, STLC, 5 μM) and the Cdk1 inhibitor (RO-3306, 1 μM).^37^ We performed a time course and dose response with the STLC/RO cocktail to determine the most effective combination for HeLa cells in our hands and settled on the 5/1 μM for 5 hours as it produced a significant mitotic arrest as judged by DAPI staining. The cells were then loaded for 30 min at room temperature using 2 μM Calbryte 590 (AAT Bioquest) to measure cytosolic Ca^2+^, they were then washed in a Ca^2+^-free solution containing 4 μM of Hoechst to stain the nuclei and identify mitotic cells. Images were collected using a Zeiss LSM 880 confocal microscope fitted with a 40x/1.3 objective with the pinhole fully open using the following parameters: for CEPIA2*mt*, λ_ex_=488 nm, λ_em_=493-556 and for Calbryte 590 λ_ex_=561nm, λ_e_λ_e_m=566-697. Images of interphase and mitotic HeLa cells were obtained using the AiryScan detector of the same microscope in Super-Resolution (SR) mode. The release of Ca^2+^ from the ER was stimulated by applying a final concentration of 100 μM histamine in the recording chamber in a Ca^2+^-free extracellular media. Images were acquired at a frame rate of 0.1 Hz. The extracellular media contained (in mM): NaCl 150, KCl 5, CaCl_2_ 1.5, D-glucose 10, MgCl_2_ 1.5 HEPES 20, pH 7.4. Image analysis was performed using Image J.

### Quantification and statistical analysis

Data are presented as mean ± SEM. Ca^2+^ and NAD(P)H kinetics were measured using Clampfit 10 (Molecular devices), outliers were removed using Prism ROUT procedure. Groups were compared using the Prism 9 software (GraphPad) using the statistical tests indicated in the figure legend. Statistical significance is indicated by p values in the Figure legends.
